# Biomarkers for predicting CAR-T cell therapy outcomes in B-cell acute lymphoblastic leukemia: a systematic review

**DOI:** 10.3389/fimmu.2025.1656108

**Published:** 2025-10-16

**Authors:** Yanhao Ke, Fen Zhou

**Affiliations:** Department of Paediatric Hematology and Oncology, Union Hospital, Tongji Medical College, Huazhong University of Science and Technology, Wuhan, Hubei, China

**Keywords:** CAR-T cell therapy, B-cell acute lymphoblastic leukemia, biomarkers, cytokinerelease syndrome, minimal residual disease, tumor burden, systematic review

## Abstract

**Introduction:**

Chimeric antigen receptor (CAR) T-cell therapy has revolutionized the treatment of relapsed/refractory B-cell acute lymphoblastic leukemia (B-ALL), yet challenges such as cytokine release syndrome (CRS), neurotoxicity (ICANS), and variable long-term efficacy persist. This systematic review evaluates the role of biomarkers in predicting CAR-T therapy outcomes, toxicity risks, and guiding personalized treatment strategies.

**Methods:**

Following PRISMA guidelines, we systematically searched PubMed, Web of Science, and Embase for studies published between 2018–2024. A total of 33 studies involving 2,095 patients were included in the analysis.

**Results:**

Key findings identified tumor burden and minimal residual disease (MRD) as dual-predictive biomarkers. High tumor burden (≥40% blasts) correlated with reduced complete remission rates (87% vs. 100%) and increased CRS/ICANS risks, while MRD negativity (NGS threshold <10⁻⁶) predicted superior 2-year event-free survival (68% vs. 23%). CAR-T functional parameters, including PD-1/LAG-3 expression (>5.2% in CD4+ cells) and peak expansion kinetics, linked efficacy to toxicity trade-offs. Genetic biomarkers (IKZF1 mutations, complex karyotypes) and biochemical indicators (m-EASIX >6.2, ferritin ≥10,000 ng/mL) further stratified risks. Unidirectional efficacy biomarkers included T-cell subsets (e.g., CD8+ naive T cells) and B-cell aplasia, while IL-6 dynamics specifically predicted CRS severity.

**Discussion:**

Despite promising insights, heterogeneity in toxicity grading systems, inconsistent biomarker thresholds, and retrospective study designs limit clinical standardization. Future directions emphasize cytoreductive bridging therapies, biomarker-guided combinatorial approaches (e.g., MDM2 inhibitors for TP53 mutations), and multicenter validation of integrated predictive models to optimize personalized CAR-T therapy strategies.

## 1 Introduction

B-cell acute lymphoblastic leukemia (B-ALL), a heterogeneous hematologic malignancy, exhibits diverse biological characteristics and variable clinical outcomes. Although intensive chemotherapy demonstrates proven efficacy in pediatric patients (1), disease relapse and poor prognosis in adults remain formidable challenges (2, 3).

Addressing these limitations, chimeric antigen receptor (CAR) T-cell therapy has fundamentally reshaped the therapeutic landscape. This innovative strategy involves genetically modifying a patient’s autologous T cells to target CD19-positive malignant cells (4), achieving remarkable efficacy: approximately 80% of relapsed/refractory patients attained complete remission in clinical trials of anti-CD19 CAR-T cell therapy (5). However, significant therapeutic hurdles persist, including cytokine release syndrome (CRS), neurotoxicity (ICANS), and concerns regarding long-term efficacy (6, 7).

Consequently, biomarkers—objectively measurable biological characteristics predicting therapeutic responses or adverse events via standardized assays (8)—have become critical for therapy optimization. Disease burden metrics and inflammatory mediators are increasingly recognized for predicting toxicity risks post CD19-targeted CAR T-cell therapy, especially CRS (9). Concurrently, cytokine dynamics and blood-brain barrier biomarkers provide crucial insights into neuroinflammatory complications (10). Integrating molecular profiling (e.g., leukemogenic driver mutations) with functional immune signatures further refines outcome prediction, though clinical validation and threshold harmonization challenges endure (11–13). This systematic review examines how CAR-T therapy-related biomarkers can predict treatment outcomes, evaluate toxicity risks, and guide personalized therapeutic strategies to optimize clinical applications in B-ALL patients.

## 2 Methods

### 2.1 Search strategy

This study followed the PRISMA guidelines (14) strictly and adopted established literature searching processes for systematic reviews (15), though it was not registered. We searched literature published between January 1, 2018, and November 8, 2024, as this period followed the significant increase in research following the FDA approval of Kymriah for relapsed/refractory B-ALL treatment in 2017 (16), which marked the formal entry of CAR-T cell therapy into clinical practice. Research published since 2018 has tracked the progression of CAR-T cell therapy from clinical trials to routine practice (17). Subsequent studies have systematically examined treatment optimization, safety protocols, and long-term outcomes, reporting key metrics like complete remission and minimal residual disease negativity rates to assess contemporary B-ALL management (18).

Our literature search employed three databases: PubMed, Web of Science, and Embase. The search strategy combined Medical Subject Headings (MeSH) terms and free-text keywords, focusing on four core concepts: CAR-T therapy (“CAR-T OR CAR T OR chimeric antigen receptor T”), biomarkers (“biomarker* OR predict* OR prognosis*”), treatment outcomes (“response OR efficacy”), and target disease (“B-ALL OR B-cell acute lymphoblastic leukemia OR B-lineage acute lymphoblastic leukemia”). Detailed search strategies for each database are provided in **Supplementary Material S2**.

### 2.2 Study selection criteria

Inclusion criteria required studies: (1) on B-ALL patients undergoing CAR-T therapy investigating biomarker associations with efficacy or toxicity; (2) randomized trials, observational studies (prospective or retrospective), or case series (≥10 patients); (3) peer-reviewed English articles published since January 2018.

Exclusion criteria specified: (1) non-B-ALL populations or studies of therapies without CAR-T; (2) studies lacking biomarker analysis; (3) systematic reviews, meta-analyses, review articles, or conference abstracts; (4) non-English publications or inadequately translated works. Complete inclusion and exclusion criteria are detailed in **Supplementary Material S3**.

Ke YH led the study selection process, which involved screening titles and abstracts, then reviewing full texts. Zhou F was regularly consulted to resolve eligibility uncertainties. All screening decisions were documented per PRISMA guidelines.

### 2.3 Data extraction

Data extraction utilized standardized forms capturing: (1) baseline characteristics (sample size, sex/age distribution, clinical status, treatment history); (2) therapeutic protocols (CAR-T product specifications, preconditioning); (3) biomarkers (types, detection methods, thresholds); and (4) clinical outcomes (efficacy/safety endpoints). All extractions adhered to predefined classification criteria.

### 2.4 Statistical analysis and quality assessment

Significant heterogeneity prevented meaningful statistical pooling across the included studies. Study designs varied considerably, including single-arm phase I/II trials (54.5%), retrospective cohort studies (36.4%), and prospective cohort analyses (9.1%). Patient ages varied widely(4 months to 76 years). Treatment protocols differed in CAR-T product targeting (CD19 single vs. CD19/CD22 dual) and costimulatory domains (4-1BB vs. CD28). Toxicity assessment methods lacked standardization, using ASTCT (15 studies), Lee (10 studies), and Penn grading systems (8 studies). Given this substantial heterogeneity, we conducted a narrative synthesis instead of a meta-analysis.

Study quality was evaluated using design-specific tools: cohort studies underwent Newcastle-Ottawa Scale assessment (evaluating cohort selection, comparability, and outcome ascertainment), while single-arm trials were appraised via the JBI checklist for single-arm studies (assessing design, implementation, and reporting). All quality assessments strictly followed established tool guidelines, with detailed assessment criteria and individual study ratings provided in **Supplementary Material S4**.

## 3 Results

### 3.1 Study selection and characteristics

**Figure 1:**
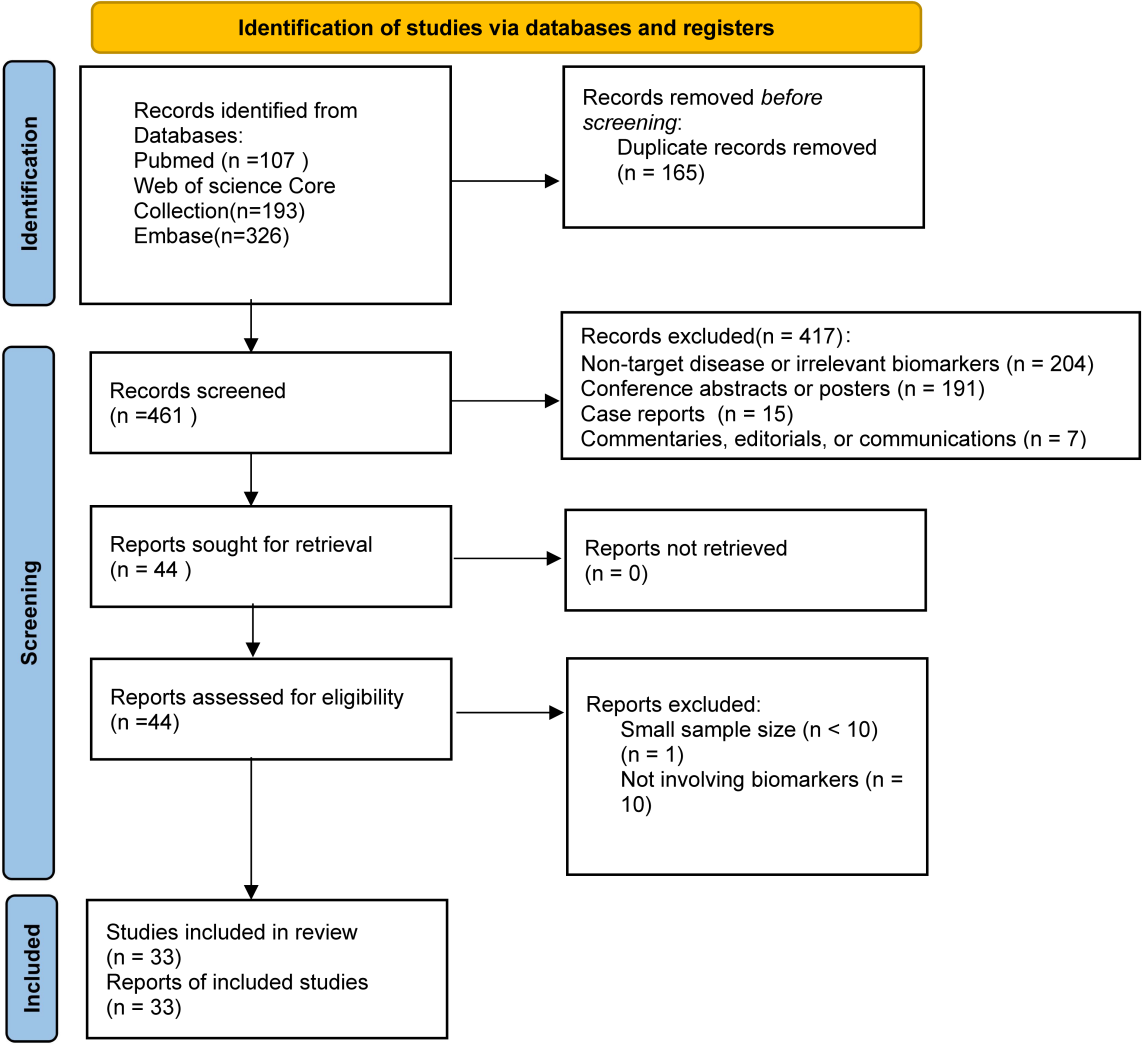
PRISMA flow diagram for study selection.

The selection process for this systematic review is shown in the PRISMA flow diagram (**Figure 1**). A total of 626 records were initially identified from three databases, and 33 studies were finally included after screening and eligibility assessment.

This systematic literature review included 33 studies (18 single-arm phase I/II trials, 12 retrospective cohort studies, and 3 prospective cohort analyses), encompassing 2,095 patients with relapsed/refractory B-ALL. Individual study populations ranged from 12 to 254 patients, with 9 studies enrolling over 100 patients, among which Zhang et al (19) reported the largest cohort (n=254). The age spectrum extended from 4 months (20) to 76 years (21). Male representation varied from 25% (22) to 74% (23). All enrolled patients had relapsed/refractory B-ALL with multiple prior lines of therapy, with some patients who had received allogeneic hematopoietic stem cell transplantation, blinatumomab, and inotuzumab.

Regarding CAR-T product characteristics, CD19 emerged as the predominant target, with products including Tisagenlecleucel, Sino19 (24–26), and AUTO1 (27). Target strategies encompassed single-targeting of CD19 or CD22, and dual-targeting of CD19/CD22 (28, 29), utilizing either 4-1BB or CD28 costimulatory domains. The standard therapeutic dose was 5×10^6 cells/kg, with total dose calculations for patients weighing over 50kg, preceded by fludarabine-cyclophosphamide conditioning regimen.

Treatment outcomes showed complete remission rates of 64.9%-100% (27 studies ≥80%) and minimal residual disease (MRD) negativity rates of 45%-100% (17 studies >80%). Safety profiles revealed CRS incidence of 55%-97.9% (grade ≥3: 10.2%-45%) and ICANS incidence of 19%-65% (grade ≥3: 2.6%-45%), evaluated using ASTCT criteria (15 studies), Lee criteria (10 studies), and Penn grading (8 studies). Long-term follow-up revealed 1-year overall survival (OS) rates of 54.8%-78.86% and progression-free survival (PFS) rates of 44.8%-69.89%. Two-year OS ranged from 59.6% to 71.4%, while 4-year OS and event-free survival (EFS) reached 69.3% and 59.0%, respectively. At 36 months, the duration of response was 56.26% (95%CI: 32.81%-74.31%) with OS of 54.72% (95%CI: 30.90%-73.38%). Notably, Next-generation sequencing (NGS) -MRD-negative patients demonstrated significantly superior 2-year EFS compared to MRD-positive patients (68%, 95%CI: 54%-86% vs 23%, 95%CI: 8.8%-62%).Detailed characteristics and outcomes for all included studies are presented in **Table 1**.

**Table 1:** Characteristics of included studies and pooled clinical outcomes.

Characteristic	Summary data
Study information	
Studies (n)	33
Total Patients (n)	2,095
Study Design (%)	Single-arm (54.5)/Retrospective (36.4)/Prospective (9.1)
Patient characteristics	
Age Range	4 months - 76 years
Disease Status	Relapsed/Refractory B-ALL (100%)
Treatment characteristics	
CAR-T Targets	CD19 (Dominant)/CD22/CD19+CD22
Cell Dose	Median 5×10^6^ cells/kg (0.5-67×10^6^)
Lymphodepletion (%)	Flu/Cy (89)/Other (11)
Response rates	
Complete Remission	64.9%-100%
MRD Negativity	45%-100%
Toxicity incidence	
CRS (All Grades)	55%-97.9%
CRS (≥Grade 3)	10.2%-45%
ICANS (All Grades)	19%-65%
ICANS (≥Grade 3)	2.6%-45%
Survival outcomes	
1-year OS	54.8%-78.86%
2-year EFS (MRD^-^ vs MRD^+^)	68% vs 23%
4-year OS	69.3%

### 3.2 Biomarker classification

**Figure 2:**
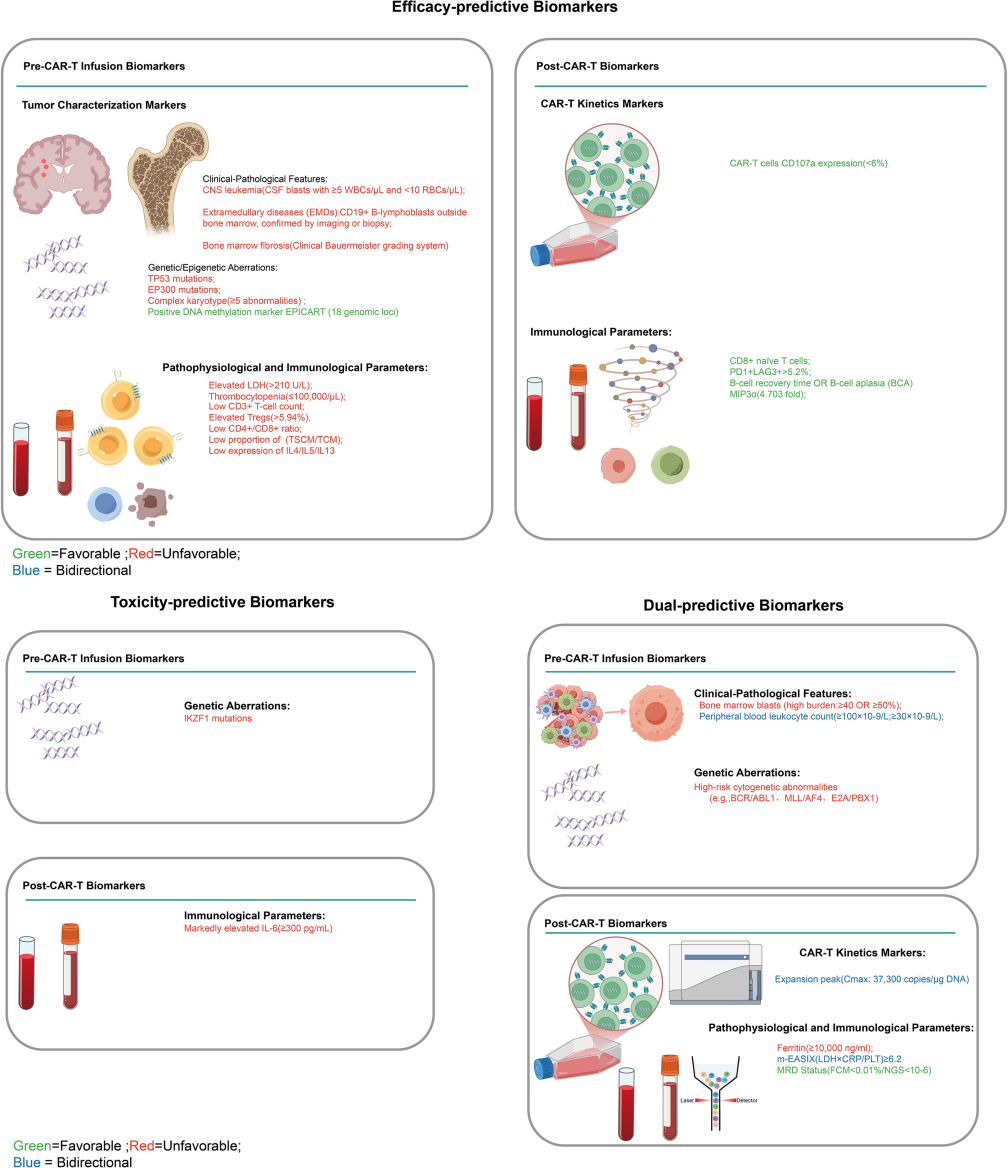
Clinical classification framework for CAR-T cell therapy biomarkers. Classification of pre-infusion and post-infusion biomarkers for CAR-T therapy efficacy and toxicity prediction. Left panel: efficacy-predictive biomarkers; Right panel: toxicity-predictive (upper) and dual-predictive biomarkers (lower). Green indicates favorable, red unfavorable, and blue bidirectional outcomes. CNS, central nervous system; EMDs, extramedullary diseases; BCA, B-cell aplasia; MRD, minimal residual disease.

The 24 biomarkers identified across studies were classified into three functional groups based on their predictive associations with treatment effects and toxicity outcomes, addressing the benefit-risk interplay in CAR-T therapy. Dual-predictive biomarkers (n=10) concurrently predicted both therapeutic effects and toxicity risks, with some markers indicating favorable efficacy outcomes alongside increased toxicity risk, while others predict poor efficacy outcomes coupled with lower toxicity risk. Favorable biomarkers (n=12) were exclusively associated with treatment efficacy outcomes, encompassing both favorable prognostic indicators (biomarkers predicting higher complete remission rates, sustained clinical benefits, and improved survival, such as CD8+ naive T cells, low Tregs ≤5.94%, PD-1+LAG-3+ expression >5.2%, B-cell aplasia, EPICART, low LDH ≤210 U/L, high platelets ≥100,000/mL, MIP3α elevation, and TH2 cytokines) and unfavorable prognostic indicators (biomarkers predicting reduced survival and poor therapeutic response, such as TP53 mutations, EP300 mutations, elevated LDH >210 U/L, low platelets, and severe bone marrow fibrosis grade ≥3), without significant toxicity linkages. Unfavorable biomarkers (n=2) were exclusively associated with toxicity manifestations and adverse event profiles (IL-6 and IKZF1 mutations), independent of therapeutic effects. The comprehensive classification framework is illustrated in **Figure 2**.

#### 3.2.1 Dual-predictive biomarkers

Tumor burden emerged as the predominant dual-predictive indicator across studies (19, 28, 30–41), defined as systemic leukemia infiltration measurable through multiple parameters: bone marrow blast percentage (thresholds: ≥50% (30–32), ≥5% (31, 35, 36, 38, 41), or >20% (19)), peripheral blood leukocyte count (≥100×10^9^/L (32)), CNS involvement (CSF blasts with ≥5 WBCs/μL and <10 RBCs/μL), and extramedullary disease (EMD) (imaging/biopsy-confirmed B lymphoblastic cells infiltration).High tumor burden consistently predicted reduced complete remission rates (87% vs. 100% with low burden (34)), increased CRS/ICANS risks (31, 32, 35), and poor long-term survival. Mechanistically, it promotes T-cell exhaustion and immunosuppressive microenvironments (42), while bone marrow blasts >20% predicted both poor outcomes and increased neurotoxicity (19).

MRD status represents another key predictor. NGS analysis showed significantly lower 2-year event-free survival in patients with MRD≥10^-6^ (23%; 95% CI: 8.8%-62%) versus MRD-negative counterparts (68%; 95% CI: 54%-86%) (43). MRD≥1% consistently correlated with poor prognosis (30, 39–41, 44) and increased CRS/ICANS risk (39, 40, 45). Day 28 MRD assessment proved clinically significant, with MRD-negative patients achieving 80.9% 2-year survival (44).

CAR-T expansion kinetics indicated peak proliferation at median day 11 post-infusion (range: 4–22 days). Higher peak levels correlated with both improved efficacy and increased toxicity risk (41).

Biochemical indicators offered additional predictive value. The modified EASIX (m-EASIX) score (LDH×CRP/platelets) served as a significant prognostic tool with dual predictive capacity. In terms of efficacy, elevated m-EASIX levels were significantly associated with reduced complete remission rates (OR 0.81, 95% CI 0.69–0.93; P = .004 at day 1 post-infusion). For toxicity prediction, an m-EASIX cutoff value >6.2 at lymphodepletion initiation effectively predicted grade ≥3 cytokine release syndrome, achieving a negative predictive value of 96.43% (23). Serum ferritin ≥10,000 ng/ml correlated with significantly reduced 1-year survival (45% vs. 100%) and functioned as a CRS predictor (22, 28).

High-risk genetic abnormalities - including complex karyotypes (≥5 aberrations) and high-risk fusion genes (e.g., BCR/ABL1, MLL/AF4, E2A/PBX1) - impacted long-term survival in CAR-T recipients despite unaffected initial remission rates. Patients with complex cytogenetics showed reduced 2-year leukemia-free survival (48.8% vs. 67.3%; p=0.039), while those with high-risk genetic/molecular abnormalities showed decreased 1-year overall survival (34.3% vs. 66.7%, p=0.047). Additionally, complex cytogenetics are linked to increased severe cytokine release syndrome (CRS) incidence (16.3% vs. 4.6%, p=0.003) (19, 24).

#### 3.2.2 Favorable biomarkers

Immune cell profiling identified critical biomarkers of therapeutic efficacy. Analysis of regulatory T cell populations revealed that patients with CD4+CD25+CD127low regulatory T cells comprising >5.94% of total CD4+ T cells demonstrated inferior outcomes, with 1-year overall survival and relapse-free survival rates of 29.3% and 11.9%, respectively, compared to 64.2% and 56.7% in patients with ≤5.94% regulatory T cells (24). Flow cytometry revealed changes in these CD4+CD25+CD127low Treg populations from 11.54% pre-treatment to 13.56% post-treatment, though this difference was not statistically significant. When patients were stratified using post-infusion Treg levels as a cutoff, those with elevated levels showed markedly poorer survival outcomes (median RFS: 64 vs 434 days, P = 0.022; median OS: 222 vs 852 days, P = 0.017) (26).

In contrast, higher proportions of CD8+ naive T cells correlated with durable remission, achieving a median OS of 12.91 months (95% CI: 7.74-18.08) (46). Similarly, memory T cell composition proved predictive, as patients with lower early memory T-cell proportions showed significantly shorter PFS (median 9.6 vs >54 months) (47), aligning with central memory T-cells’ established role in CAR-T expansion and persistence (48). Internal and external validation further established clinically relevant CD3+ T cell count thresholds at 0.973×10^9/L for predicting CAR-T therapy response and 0.723×10^9/L for predicting therapy success, with corresponding CD4+T/CD8+T ratio thresholds of 0.744 and 0.887 that predicted improved treatment outcomes (25).

Functional profiling revealed additional key insights. Notably, >5.2% PD-1+LAG-3+ expression in CD4+ CAR-T cells correlated with superior event-free survival (77% vs. 42%; p<0.01) (49), despite LAG-3 being frequently co-expressed with PD-1 on Tconv and Tregs where it mediates immunosuppression through MHC class II binding and promotes T-cell exhaustion (50, 51). Furthermore, reduced CD107a expression (6%) in CD8+ CAR-T cells, as measured via flow cytometry/digital PCR/ELISA, was associated with improved survival (49). Persistent B-cell aplasia (BCA) served as another reliable marker, with B-cells/leukocytes <1% or B-cells/lymphocytes <3% (37, 43, 52) strongly predicting remission and survival (27), whereas B-cell recovery within 6 months indicated poorer outcomes (43).

Among molecular biomarkers, the DNA methylation marker EPICART, encompassing 18 genomic loci, showed significant association with complete remission and prolonged EFS (HR = 0.36, P = 0.003), likely through epigenetic regulation of CAR-T persistence (53). Multivariate analysis identified key genetic alterations as independent predictors of treatment outcomes (19, 32, 44), with TP53-mutated patients showing significantly lower survival versus wild-type (1-year OS: 57.2% vs 82.3%; P = 0.03) and EP300 mutations demonstrating similar survival impairment. These findings contributed to robust clinical outcomes, with an overall MRD negativity rate post-CAR-T of 73.7% and cohort-wide 2-year OS and DFS rates of 71.4% and 60.5% respectively (44).

Pre-treatment laboratory parameters offered additional prognostic value (20, 21, 40). Patients with low LDH (≤210 U/L) and high platelets (≥100,000/μL) achieved superior EFS, while fludarabine-containing lymphodepletion improved responses, resulting in higher MRD negativity (85%) and sustained remission rates (20). The bone marrow microenvironment also influenced outcomes, with severe reticulin fibrosis (grade ≥3) correlating with poorer clinical results and a median survival of only 250 days compared to 1,463 days for lower-grade fibrosis (54). Conversely, elevated MIP3α levels (4.7-fold above healthy donors) predicted favorable progression-free survival (p=0.0049 in training cohort, p=0.0190 in validation cohort), with 67.5% of relapses occurring within six months post-infusion, possibly via CCR6-mediated T-cell infiltration and memory phenotype enrichment (29). Additionally, TH2-associated cytokines (IL4, IL5, IL13) predicted complete remission rates, while functional impairment correlated with increased CD19+ relapse risk (47).

#### 3.2.3 Unfavorable biomarkers

Among the evaluated biomarkers, IL-6 emerged as a key unidirectional toxicity predictor exclusively associated with adverse event manifestations independent of therapeutic effects (28, 55). IL-6 dynamics analysis showed day 7 post-CAR-T levels specifically correlated with cytokine release syndrome (CRS) development but demonstrated no association with efficacy outcomes. Beyond IL-6, comprehensive biomarker evaluation revealed additional cytokines with significant predictive value for toxicity manifestations, though IL-6 and the genetic marker IKZF1 (discussed below) constitute the primary independent predictors with robust clinical evidence. In pediatric populations, sgp130 and MCP-1 showed significant association with CRS development (9). Furthermore, peak serum levels of IL-8, IL-10, IL-15, interferon gamma, and TNF receptor p55 within 36 hours post-infusion were associated with severe CRS manifestations.

For neurotoxicity prediction, patients with high IL-15 levels (≥50 pg/mL) combined with low EGF levels (<120 pg/mL) demonstrated 100% risk of severe neurotoxicity, while those with low IL-15 or high EGF had only 11% risk of developing this adverse event. Additionally, elevated angiopoietin-2 to angiopoietin-1 ratios correlated with severe neurotoxicity (10). Clinical assessment revealed that CRS occurred in 55.3% of patients, including 13.2% with severe (grade ≥3) events (55).

**Figure 3:**
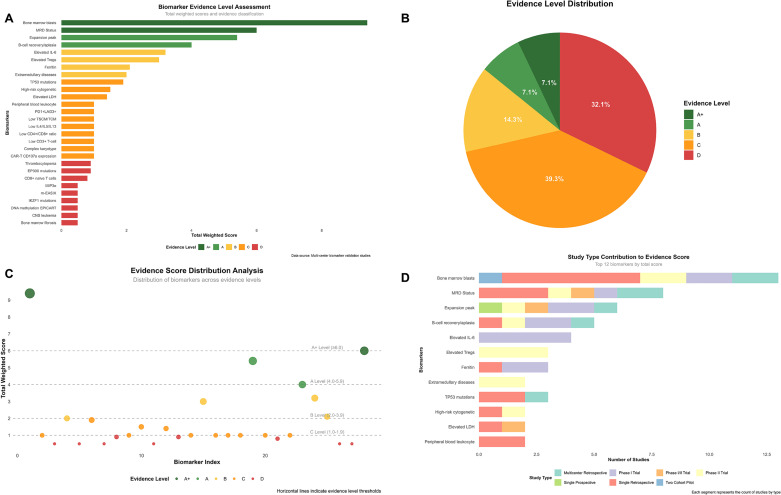
Comprehensive evidence analysis of CAR-T biomarkers. Evidence assessment based on standardized weighted scoring system with research type coefficients: multicenter retrospective cohort (0.9), single-arm phase II trial (1.0), single-arm phase I trial (0.8), single-arm phase I/II trial (0.9), single-center prospective cohort (1.0), single-center retrospective cohort (0.5), prospective two-cohort pilot study (1.0). Evidence levels defined as: A+ (≥6.0, top-tier evidence), A (4.0-5.9, strong evidence), B (2.0-3.9, moderate evidence), C (1.0-1.9, weak evidence), D (<1.0, very weak evidence). **(A)** Biomarker evidence level assessment ranking based on study count and quality weighting. **(B)** Distribution of biomarkers across evidence levels (A+: 7.1%, A: 7.1%, B: 14.3%, C: 39.3%, D: 32.1%). **(C)** Evidence scores vs. biomarker index with level thresholds indicated by horizontal lines. **(D)** Study type contributions to evidence scores for top 12 biomarkers.

Genetic biomarkers demonstrated association with adverse events: IKZF1 mutations showed an association with increased neurotoxicity risk as identified through multivariable analysis, though specific incidence rates varied across different patient subgroups. The overall neurotoxicity incidence was 19.0% for any grade, with 5.2% experiencing severe neurotoxicity (39). To systematically evaluate the evidence strength of these biomarkers, we developed a standardized weighted scoring system (**Figure 3**).

## 4 Discussion

### 4.1 Core biomarkers and their mechanistic insights

Our systematic review identifies bone marrow tumor burden as a principal prognostic factor in B-ALL CAR-T outcomes. Patients with high tumor burden (≥40% blasts) exhibited reduced complete remission rates and long-term survival, consistent with established mechanisms of T-cell exhaustion and immunosuppressive microenvironments (42). These findings support the use of bridging therapies such as inotuzumab and blinatumomab for effective cytoreduction (56, 57).

Immune cell composition further refined prognostic stratification. A threshold of 5.94% for CD4+CD25+CD127low regulatory T cells (Tregs) within total CD4+ T cells reliably discriminated between favorable and unfavorable outcomes. Treg levels above this limit may suppress CAR-T function through IL-10 and TGF-β secretion, direct cell contact inhibition, and metabolic interference. In contrast, enriched early memory T-cell subsets (Tscm and Tcm) emerged as favorable biomarkers, demonstrating enhanced proliferative capacity, reduced exhaustion, and sustained persistence—properties critical for long-term efficacy (58). Tcm-enriched CAR-T products combined with fludarabine significantly improved persistence (p<0.01) and progression-free survival (p=0.001) (48).

Beyond biomarkers, treatment-related factors influenced outcomes. Newer-generation CAR-T products yielded superior remission rates, while dual-targeting or sequential infusion strategies achieved higher MRD negativity (32). These findings indicate sequential infusion of CD19/CD22 multi-targeted CAR-T cells may offer promising personalized approaches for refractory/relapsed B-ALL. While CD19 expression intensity lacked prognostic impact, prior blinatumomab exposure potentially compromised CAR-T efficacy (56, 59).

### 4.2 Cytokine profiles and integrated predictive models

Cytokine monitoring post-infusion enhances predictive accuracy for both toxicity and response. Beyond IL-6, which remains indicative of severe cytokine release syndrome (CRS) at levels exceeding 1000 pg/mL, a multi-cytokine panel (IL-8, IL-10, IL-15, IFN-γ, TNF receptor p55) within 36 hours improved prediction of severe CRS. The combination of IL-15 ≥50 pg/mL and EGF <120 pg/mL specifically identified patients at 100% risk of severe neurotoxicity, supporting preemptive management in high-risk cases.

A TH2-oriented cytokine profile (IL-4, IL-5, IL-13) correlated with complete remission and prolonged survival, suggesting a protective immunologic milieu. Integrative models combining cellular and cytokine variables—such as CAR+TH2+ frequency and memory T-cell counts—achieved 70% sensitivity with less than 5% false-positive rates, indicating strong potential for clinical translation (26).

### 4.3 Molecular biomarkers and novel therapeutic targets

Somatic mutations create critical therapeutic windows in cancer treatment. TP53 mutations, among the most significant alterations, disrupt normal tumor suppression mechanisms (60). This vulnerability has led to the development of MDM2 inhibitors, which work by preventing MDM2-mediated p53 degradation. APG-115 demonstrates this strategy, effectively activating p53 and p21 while boosting antitumor immunity. The strategy proves most effective when TP53 remains functionally intact, allowing restored p53 signaling to eliminate malignant cells (61).

EP300 dysfunction presents another compelling therapeutic target in acute lymphoblastic leukemia. The EP300-ZNF384 fusion protein drives B-ALL progression through aberrant activation of genes like IL3RA (62). Unlike conventional approaches, targeting this epigenetic dysregulation offers a more precise intervention. Early studies suggest that disrupting EP300-mediated transcription can reactivate silenced tumor suppressors and improve treatment responses, particularly in fusion-positive cases where normal epigenetic control has been compromised.

Epigenetic regulation contributed to CAR-T therapy efficacy through the EPICART methylation signature, which affected gene expression patterns in responding versus non-responding patients (53). Translationally, pre-infusion CD3+ T-cell counts and CD4+/CD8+ ratios offered actionable thresholds for patient stratification (25), while achievement of minimal residual disease negativity by day 28 emerged as a critical early indicator of long-term leukemia-free survival (30).

### 4.4 Standardization challenges and technical advances

Dynamic MRD monitoring is pivotal for outcome prediction in CAR-T therapy. Our findings confirm that day 28 MRD status serves as a critical prognostic marker, with MRD-negative patients achieving significantly superior 2-year event-free survival compared to those with MRD≥10^-6^ (68% vs 23%) (43, 44). Higher MRD burdens (≥1%) consistently correlate with both poor clinical outcomes and increased toxicity risks (30, 39–41, 44, 45). Standardizing detection methods (NGS vs flow cytometry) and thresholds (10^-4^ vs 10^-6^) is critical for cross-study harmonization (11, 13). Digital PCR (dPCR) has emerged as a superior tool for CAR-T copy quantification due to high sensitivity and absolute quantification without standard curves. While dPCR, NGS, and flow cytometry all detect MRD, dPCR offers distinct advantages for low-frequency MRD detection (63).

CD19 dynamics and prior blinatumomab exposure may influence outcomes. Emerging surface markers (CD5, CD123, CD33, CD70, CD38, BCMA) and T-cell distributions show promise pending multicenter validation (59), suggesting substantial potential for improving CAR-T prediction systems (9, 64).

IL-6 and ferritin are cornerstone CRS severity predictors. IL-6 levels >1000 pg/mL indicate severe CRS risk, while daily levels >15.2 pg/mL predict grade ≥3 ICANS (9). Tocilizumab (IL-6R antagonist) suppresses CRS in ASTCT grade 2 with significant IL-6 elevation (≥4-fold increase) (65). However, administration with <4-fold IL-6 increase may elevate severe CRS risk (66, 67). Ferritin >10,000 ng/mL necessitates early intervention, predicting severe toxicity and reduced survival (45% vs 100% at 1 year) (22). The m-EASIX score (LDH×CRP/PLTs) >6.2 further refines risk stratification, enabling preemptive high-risk management (23).

Heterogeneity in toxicity grading systems (ASTCT, Lee, Penn criteria) complicates cross-study comparisons. In JULIET, CRS grading showed significant discordance: 38% downgraded (Lee vs Penn) and 36% downgraded (ASTCT vs Penn), particularly for grade 2 events (68).

### 4.5 Limitations and future directions

This review highlights several limitations affecting the current evidence. Heterogeneity in sample sizes (12–254 patients) and follow-up duration (6–48 months) may affect the generalizability of results. Inconsistent use of toxicity grading systems (e.g., ASTCT, Lee, and Penn criteria) complicates cross-trial comparisons. Furthermore, variability in biomarker assays and thresholds (e.g., tumor burden cutoffs ranging from ≥5% to ≥50%) impedes unified clinical application.

Most studies were retrospective and did not adequately control for confounding variables via multivariate modeling. Research on mutations such as TP53 and EP300 has focused predominantly on prognostic association rather than mechanistic dissection or therapeutic targeting. Similarly, although IL-6 and ferritin are established toxicity markers, intervention thresholds remain poorly defined. Most biomarkers have been studied in isolation; few analyses incorporate multi-parameter models reflective of clinical complexity.

Future studies should prioritize prospective, multi-center designs using standardized endpoints and assay methods. There is a particular need for integrative biomarker models that incorporate cellular, soluble, genetic, and clinical variables to improve risk stratification and support personalized treatment strategies.

## Data Availability

The original contributions presented in the study are included in the article/**Supplementary Material**, further inquiries can be directed to the corresponding author/s.
