# Supplementary material for: Biomarkers for predicting CAR-T cell therapy outcomes in B-cell acute lymphoblastic leukemia: a systematic review
Source: Front Immunol. 2025 Oct 16;16:1656108. doi: 10.3389/fimmu.2025.1656108 (PMC12571805; doi:10.3389/fimmu.2025.1656108)
Supplement: Supplementary file 1 [file Table1.docx]

Supplementary Material

# Supplementary Material S1: PRISMA 2020 Checklist

| **Section and Topic** | **Item #** | **Checklist item** | **Location where item is reported** |
| --- | --- | --- | --- |
| **TITLE** | | |  |
| Title | 1 | Identify the report as a systematic review. | Title contains "systematic review" |
| **ABSTRACT** | | |  |
| Abstract | 2 | See the PRISMA 2020 for Abstracts checklist. | Lines 11-30 |
| **INTRODUCTION** | | |  |
| Rationale | 3 | Describe the rationale for the review in the context of existing knowledge. | Lines 32-50 |
| Objectives | 4 | Provide an explicit statement of the objective(s) or question(s) the review addresses. | Lines 51-52 |
| **METHODS** | | |  |
| Eligibility criteria | 5 | Specify the inclusion and exclusion criteria for the review and how studies were grouped for the syntheses. | Lines 71-79; Supplementary Material S3 |
| Information sources | 6 | Specify all databases, registers, websites, organisations, reference lists and other sources searched or consulted to identify studies. Specify the date when each source was last searched or consulted. | Lines 55-64 |
| Search strategy | 7 | Present the full search strategies for all databases, registers and websites, including any filters and limits used. | Lines 64-70; Supplementary Material S2 |
| Selection process | 8 | Specify the methods used to decide whether a study met the inclusion criteria of the review, including how many reviewers screened each record and each report retrieved, whether they worked independently, and if applicable, details of automation tools used in the process. | Lines 80-82 |
| Data collection process | 9 | Specify the methods used to collect data from reports, including how many reviewers collected data from each report, whether they worked independently, any processes for obtaining or confirming data from study investigators, and if applicable, details of automation tools used in the process. | Lines 83-87 |
| Data items | 10a | List and define all outcomes for which data were sought. Specify whether all results that were compatible with each outcome domain in each study were sought (e.g. for all measures, time points, analyses), and if not, the methods used to decide which results to collect. | Lines 83-87;  Table 1 |
|  | 10b | List and define all other variables for which data were sought (e.g. participant and intervention characteristics, funding sources). Describe any assumptions made about any missing or unclear information. | Lines 83-87 |
| Study risk of bias assessment | 11 | Specify the methods used to assess risk of bias in the included studies, including details of the tool(s) used, how many reviewers assessed each study and whether they worked independently, and if applicable, details of automation tools used in the process. | Lines 97-101 |
| Effect measures | 12 | Specify for each outcome the effect measure(s) (e.g. risk ratio, mean difference) used in the synthesis or presentation of results. | Not Reported |
| Synthesis methods | 13a | Describe the processes used to decide which studies were eligible for each synthesis (e.g. tabulating the study intervention characteristics and comparing against the planned groups for each synthesis (item #5)). | Lines 95-96 |
|  | 13b | Describe any methods required to prepare the data for presentation or synthesis, such as handling of missing summary statistics, or data conversions. | Not Reported |
|  | 13c | Describe any methods used to tabulate or visually display results of individual studies and syntheses. | All Table and Figures |
|  | 13d | Describe any methods used to synthesize results and provide a rationale for the choice(s). If meta-analysis was performed, describe the model(s), method(s) to identify the presence and extent of statistical heterogeneity, and software package(s) used. | Lines 95-96;  Lines107-114 |
|  | 13e | Describe any methods used to explore possible causes of heterogeneity among study results (e.g. subgroup analysis, meta-regression). | Not applicable |
|  | 13f | Describe any sensitivity analyses conducted to assess robustness of the synthesized results. | Not Reported |
| Reporting bias assessment | 14 | Describe any methods used to assess risk of bias due to missing results in a synthesis (arising from reporting biases). | Not Reported |
| Certainty assessment | 15 | Describe any methods used to assess certainty (or confidence) in the body of evidence for an outcome. | Lines 97-101 |
| **RESULTS** | | |  |
| Study selection | 16a | Describe the results of the search and selection process, from the number of records identified in the search to the number of studies included in the review, ideally using a flow diagram. | Lines 103-106; Figure 1 |
|  | 16b | Cite studies that might appear to meet the inclusion criteria, but which were excluded, and explain why they were excluded. | Not Reported |
| Study characteristics | 17 | Cite each included study and present its characteristics. | Lines 107-132; Table 1 |
| Risk of bias in studies | 18 | Present assessments of risk of bias for each included study. | Supplementary Material S4 |
| Results of individual studies | 19 | For all outcomes, present, for each study: (a) summary statistics for each group (where appropriate) and (b) an effect estimate and its precision (e.g. confidence/credible interval), ideally using structured tables or plots. | Lines 133-224 |
| Results of syntheses | 20a | For each synthesis, briefly summarise the characteristics and risk of bias among contributing studies. | Lines 107-132; Table 1 |
|  | 20b | Present results of all statistical syntheses conducted. If meta-analysis was done, present for each the summary estimate and its precision (e.g. confidence/credible interval) and measures of statistical heterogeneity. If comparing groups, describe the direction of the effect. | Not applicable |
|  | 20c | Present results of all investigations of possible causes of heterogeneity among study results. | Not applicable |
|  | 20d | Present results of all sensitivity analyses conducted to assess the robustness of the synthesized results. | Not applicable |
| Reporting biases | 21 | Present assessments of risk of bias due to missing results (arising from reporting biases) for each synthesis assessed. | Not applicable |
| Certainty of evidence | 22 | Present assessments of certainty (or confidence) in the body of evidence for each outcome assessed. | Figure 3 |
| **DISCUSSION** | | |  |
| Discussion | 23a | Provide a general interpretation of the results in the context of other evidence. | Lines 226-281 |
|  | 23b | Discuss any limitations of the evidence included in the review. | Lines 282-298 |
|  | 23c | Discuss any limitations of the review processes used. | Lines 282-298 |
|  | 23d | Discuss implications of the results for practice, policy, and future research. | Lines 282-298 |
| **OTHER INFORMATION** | | |  |
| Registration and protocol | 24a | Provide registration information for the review, including register name and registration number, or state that the review was not registered. | Lines 55-56 |
|  | 24b | Indicate where the review protocol can be accessed, or state that a protocol was not prepared. | Not Reported |
|  | 24c | Describe and explain any amendments to information provided at registration or in the protocol. | Not Reported |
| Support | 25 | Describe sources of financial or non-financial support for the review, and the role of the funders or sponsors in the review. | Lines 522 |
| Competing interests | 26 | Declare any competing interests of review authors. | Lines 511-513 |
| Availability of data, code and other materials | 27 | Report which of the following are publicly available and where they can be found: template data collection forms; data extracted from included studies; data used for all analyses; analytic code; any other materials used in the review. | Lines 527-532 |

# Supplementary Material S2: Detailed Search Strategies by Database

| Database | （2018.1.1-2024.11.8） |
| --- | --- |
| PubMed | (CAR-T OR CAR T OR chimeric antigen receptor T) AND (biomarker* OR predict* OR prognos*) AND (response OR efficacy) AND (B-ALL OR B-cell acute lymphoblastic leukemia OR B-lineage acute lymphoblastic leukemia)NOT (review[Publication Type] OR systematic review[Publication Type] OR meta-analysis[Publication Type] OR case reports[Publication Type] OR comment[Publication Type]) |
| Web of Science | TS=((CAR-T OR CAR T OR chimeric antigen receptor T) AND (biomarker* OR predict* OR prognos*) AND (response OR efficacy) AND (B-ALL OR B-cell acute lymphoblastic leukemia OR B-lineage acute lymphoblastic leukemia) ) NOT (DT=(Review OR "Systematic Review" OR "Meta-Analysis" OR "Case Report" OR Comment)) |
| Embase | (CD19:ti,ab OR "CD-19":ti,ab) AND ("CAR-T":ti,ab OR "CAR T":ti,ab OR "chimeric antigen receptor T":ti,ab) AND (biomarker*:ti,ab OR predict*:ti,ab OR prognos*:ti,ab) AND (response:ti,ab OR efficacy:ti,ab OR toxicit*:ti,ab OR "adverse event*":ti,ab OR "side effect*":ti,ab) AND (pediatric*:ti,ab OR child*:ti,ab OR adolescent*:ti,ab OR young*:ti,ab) AND ("B-ALL":ti,ab OR "B-cell acute lymphoblastic leukemia":ti,ab OR "B-lineage acute lymphoblastic leukemia":ti,ab) NOT ('review'/it OR 'systematic review'/it OR 'meta analysis'/it OR 'case report'/it OR 'comment'/it) |

Common search term combinations for PubMed, Web of Science, and OVID-Embase:
(CAR-T OR CAR T OR chimeric antigen receptor T) AND (biomarker* OR predict* OR prognos*) AND (response OR efficacy) AND (B-ALL OR B-cell acute lymphoblastic leukemia OR B-lineage acute lymphoblastic leukemia)（2018.1.1-2024.11.8）

# Supplementary Material S3: Inclusion and Exclusion Criteria

We screened the literature according to the following inclusion and exclusion criteria to ensure studies met the objectives of the systematic review:

**Inclusion Criteria:**

1.Study population consists of patients with acute B-cell lymphoblastic leukemia (B-ALL).

2.Patients received CAR-T cell therapy (no restriction on CAR-T targeting).

3.Studies provided data or analysis on the relationship between biomarkers and efficacy (such as complete remission rate, MRD status, survival rate) or toxicity (such as CRS, neurotoxicity).

4.Literature types include randomized controlled trials (RCTs), observational studies (such as cohort studies, case-control studies), and case series (n≥10).

5.Peer-reviewed articles published from January 2018 to present in English.

**Exclusion Criteria:**

1.Non-B-ALL patients, or patients with other types of leukemia (such as T-ALL or AML) or other malignancies.

2.Patients who did not receive CAR-T therapy, or only received other treatments (such as allo-HSCT, chemotherapy).

3.Studies that did not provide biomarker-related data or analysis.

4.Literature types including systematic literature reviews (SLR), meta-analyses, review articles, letters, conference abstracts, preprints, and case reports (n<10).

5.Non-English literature (if translation is unavailable) or literature with poor translation quality.

# Supplementary Material S4: Detailed Quality Assessment Results

JBI Case Series Scale

| Literature Information | Q1 | Q2 | Q3 | Q4 | Q5 | Q6 | Q7 | Q8 | Q9 | Q10 | Quality Assessment |
| --- | --- | --- | --- | --- | --- | --- | --- | --- | --- | --- | --- |
| An et al. 2020 (39) | Yes | Yes | Yes | Yes | No | Yes | Yes | Yes | Yes | Yes | High Quality |
| Gong et al. 2022 (20) | Yes | Yes | Yes | Yes | Yes | Yes | Yes | Yes | Yes | Yes | High Quality |
| Gu et al., 2020 (40) | Yes | Yes | Yes | Yes | Yes | Yes | Yes | Yes | Yes | Yes | High Quality |
| Hay et al.2019(52) | Yes | Yes | Yes | Yes | Yes | Yes | Yes | Yes | Yes | Yes | High Quality |
| Jacoby et al.2022 (22) | Yes | Yes | Yes | Yes | Yes | Yes | Yes | Yes | Yes | Yes | High Quality |
| Li et al., 2022 (25) | Yes | Yes | Yes | Yes | Yes | Yes | Yes | Yes | Yes | Yes | High Quality |
| Lu et al., 2021 (36) | Yes | Yes | Yes | Yes | Yes | Yes | Yes | Yes | Yes | Yes | High Quality |
| Mueller et al., 2018 (48) | Yes | Yes | Yes | Yes | Yes | Yes | Yes | Yes | Yes | Yes | High Quality |
| Myers et al., 2021 (26) | Yes | Yes | Yes | Yes | Yes | Yes | Yes | Yes | Yes | Yes | High Quality |
| Ortíz-Maldonado et al., 2021 (55) | Yes | Yes | Yes | Yes | Yes | Yes | Yes | Yes | Yes | Yes | High Quality |
| Pan et al., 2021 (42) | Yes | Yes | Yes | Yes | Yes | Yes | Yes | Yes | Yes | Yes | High Quality |
| Pu et al., 2024 (41) | Yes | Yes | Yes | Yes | Yes | Yes | Yes | Yes | Yes | Yes | High Quality |
| Roddie et al. 2021 (49) | Yes | Yes | Yes | Yes | No | Yes | Yes | Yes | Yes | Yes | High Quality |
| Wang et al. 2020 (30) | Yes | Yes | Yes | Yes | Yes | Yes | Yes | Yes | Yes | Yes | High Quality |
| Wang et al. 2024 (31) | Yes | Yes | Yes | Yes | Yes | Yes | Yes | Yes | Yes | Yes | High Quality |

JBI Case Series Scale Questions:

Q1: Clear inclusion criteria?

Q2: Standard, reliable condition measurement?

Q3: Valid methods for condition identification?

Q4: Consecutive inclusion of participants?

Q5: Complete inclusion of participants?

Q6: Clear demographic information?

Q7: Clear clinical information?

Q8: Clear outcome/follow-up reporting?

Q9: Clear site demographic information?

Q10: Appropriate statistical analysis?

NOS Cohort Study Scale

| Literature Information | Q1 | Q2 | Q3 | Q4 | Q5 | Q6 | Q7 | Q8 | Q9 | Quality Grade |
| --- | --- | --- | --- | --- | --- | --- | --- | --- | --- | --- |
| Anil et al. 2023 (53) | Yes | Yes | Yes | Yes | Yes | Yes | Yes | Yes | Yes | 9★ (High Quality) |
| Bai et al. 2022 (43) | Yes | Yes | Yes | Yes | Yes | Yes | Yes | Yes | Yes | 9★ (High Quality) |
| Dourthe et al. 2021 (19) | Yes | Yes | Yes | Yes | Yes | Yes | Yes | Yes | Yes | 9★ (High Quality) |
| García-Calderón et al. 2023 (45) | Yes | Yes | Yes | Yes | Yes | Yes | Yes | Yes | Yes | 9★ (High Quality) |
| Garcia-Prieto et al. 2022 (50) | Yes | Yes | Yes | Yes | Yes | Yes | Yes | Yes | Yes | 9★ (High Quality) |
| Gu et al. 2022 (21) | Yes | Yes | Yes | Yes | Yes | Yes | Yes | Yes | Yes | 9★ (High Quality) |
| Jin et al. 2023 (54) | Yes | No | Yes | Yes | Yes | Yes | Yes | Yes | Yes | 8★ (High Quality) |
| Kadauke et al. 2021 (23) | Yes | Yes | Yes | Yes | Yes | Yes | No | Yes | Yes | 8★ (High Quality) |
| Li et al., 2020 (24) | Yes | Yes | Yes | Yes | Yes | Yes | No | Yes | Yes | 8★ (High Quality) |
| Li et al., 2022 (35) | Yes | Yes | Yes | Yes | Yes | Yes | No | Yes | Yes | 8★ (High Quality) |
| Pennisi et al., 2021 (37) | Yes | Yes | Yes | Yes | Yes | Yes | No | Yes | Yes | 8★ (High Quality) |
| Pillai et al., 2019 (58) | Yes | Yes | Yes | Yes | Yes | Yes | Yes | Yes | Yes | 8★ (High Quality) |
| Pulsipher et al., 2022 (34) | Yes | Yes | Yes | Yes | Yes | Yes | Yes | Yes | Yes | 9★ (High Quality) |
| Schultz et al. 2022 (27) | Yes | Yes | Yes | Yes | Yes | Yes | Yes | Yes | Yes | 9★ (High Quality) |
| Shang et al. 2024 (28) | Yes | Yes | Yes | Yes | Yes | Yes | Yes | Yes | Yes | 9★ (High Quality) |
| Shang et al. 2024 (29) | Yes | Yes | Yes | Yes | Yes | Yes | Yes | Yes | Yes | 9★ (High Quality) |
| Zhang et al. 2022 (32) | Yes | Yes | Yes | Yes | Yes | Yes | Yes | Yes | Yes | 9★ (High Quality) |
| Zhou et al. 2022 (38) | Yes | Yes | Yes | Yes | Yes | Yes | Yes | Yes | Yes | 9★ (High Quality) |

NOS Cohort Study Scale Questions:

Q1: Representativeness of the exposed cohort?

Q2: Selection of the non-exposed cohort from the same source?

Q3: Reliable exposure assessment?

Q4: Outcome not present at study start?

Q5: Design/analysis controls for key factors?

Q6: Controls for additional factors?

Q7: Blind outcome assessment?

Q8: Adequate follow-up period?

Q9: Completeness of follow-up (no significant bias)?
